# Conventional Mirror Therapy versus Immersive Virtual Reality Mirror Therapy: The Perceived Usability after Stroke

**DOI:** 10.1155/2023/5080699

**Published:** 2023-05-25

**Authors:** Eliana da Silva Jaques, Anelise Ineu Figueiredo, Aniuska Schiavo, Bianca Pacheco Loss, Gabriel Hoff da Silveira, Vicenzo Abichequer Sangalli, Denizar Alberto da Silva Melo, Léder Leal Xavier, Márcio Sarroglia Pinho, Régis Gemerasca Mestriner

**Affiliations:** ^1^Biomedical Gerontology Program of the School of Medicine, Pontifical Catholic University of Rio Grande do Sul (PUCRS), Porto Alegre, Brazil; ^2^Neuroplasticity and Rehabilitation Research Group (NEUROPLAR), Pontifical Catholic University of Rio Grande do Sul (PUCRS), Porto Alegre, Brazil; ^3^School of Health and Life Sciences, Pontifical Catholic University of Rio Grande do Sul (PUCRS), Porto Alegre, Brazil; ^4^Pontifical Catholic University of Rio Grande do Sul (PUCRS), Polytechnic School, Virtual Reality Research Group, Porto Alegre, Brazil

## Abstract

**Background:**

Stroke is a widespread and complex health issue, with many survivors requiring long-term rehabilitation due to upper-limb impairment. This study is aimed at comparing the perceived usability of two feedback-based stroke therapies: conventional mirror therapy (MT) and immersive virtual reality mirror therapy (VR).

**Methods:**

The study involved 45 participants, divided into three groups: the stroke survivors (*n* = 15), stroke-free older adults (*n* = 15), and young controls (*n* = 15). Participants performed two tasks using both MT and VR in a semirandom sequence. Usability instruments (SUS and NASA-TLX) were applied at the end of the activities, along with two experience-related questions.

**Results:**

The results indicated that both MT and VR had similar levels of perceived usability, with MT being more adaptable and causing less overall discomfort. Conversely, VR increased the perception of task difficulty and prevented participants from diverting their attention from the mirror-based feedback.

**Conclusion:**

While VR was found to be less comfortable than MT, both systems exhibited similar perceived usability. The comfort levels of the goggles may play a crucial role in determining the usability of VR for upper limb rehabilitation after stroke.

## 1. Introduction

Stroke is a major global health problem that results in high acute and chronic care-related costs and contributes to the overall burden of disease [1, 2]. Upper-limb impairment is a common consequence of stroke, with up to 60% of survivors requiring long-term rehabilitation [3–5]. Virtual reality (VR) has been increasingly used in neurorehabilitation to provide lifelike environments. Its application has been studied in various neurological conditions such as stroke, Parkinson's disease, cerebral palsy, and multiple sclerosis [6–8]. Immersive virtual reality mirror therapy (VR), which replaces physical mirrors used in conventional mirror therapy (MT), has been proposed to increase the therapeutic value of mirror therapy [9].

Mirror neurons, located in the premotor cortex of the frontal lobe, are activated both when an individual performs movements and when they observe others moving [10]. However, conventional MT has several disadvantages that limit its use in clinical settings, including: (a) a monotonous and low-dose therapy; (b) the need for dedicated apparatus and often a professional on site; (c) the need for the patient to constantly observe the mirror-generated feedback; and (d) the potential perception of bilateral rather than unilateral movement [11]. VR-based therapy may help overcome these limitations by facilitating the application of mirror therapy principles in a more clinically feasible manner.

VR is believed to be a promising way to achieve higher doses of therapy and improve poststroke arm/hand recovery [12]. The integration of VR technology with MT principles presents an interesting approach for enhancing stroke rehabilitation outcomes, but the literature is scarce. Moreover, a thorough evaluation of the perceived usability of VR-based feedback in comparison to conventional MT using smartphones with VR capabilities has yet to be carried out. Despite the availability of VR-dedicated devices on the market that offer superior image quality and immersive experiences, at the moment, their cost remains a significant barrier to accessibility, particularly for individuals living in developing regions. Therefore, the current study was designed to address the perceived usability of MT and VR (using a smartphone with VR googles) feedback-based stroke therapies.

## 2. Material and Methods

This study was conducted at an outpatient rehabilitation center. Some participants who were unable to attend the facility were assessed in their homes. Two trained physiotherapists reviewed the inclusion and exclusion criteria and performed data collection.

### 2.1. Participants

We recruited 15 participants for each group, as typically used in phase I (exploratory) usability trials [13]. Thus, 45 participants were recruited by convenience to establish the following groups: stroke (*n* = 15), stroke-free older adults (*n* = 15), and young participants (*n* = 15), the latter two constituting controls. The stroke group (*n* = 15) included participants aged between 30 and 80 years old with stroke-related arm/hand deficits according to the Fugl-Meyer assessment criteria for upper-limb function [14]. Participants with a history of comprehension aphasia, severe visual deficits, mild cognitive impairment or dementia, upper-limb amputation, or other upper-limb impairments unrelated to stroke were excluded. The stroke-free older adults (*n* = 15) included participants aged 60 to 80 years old, and the young group (*n* = 15) consisted of participants aged 18 to 35 years old, both without a history of conditions affecting upper-limb function.

### 2.2. Outcomes

The main study outcome, perceived usability, was assessed using the System Usability Scale (SUS) [15] and the NASA Task Load Index (NASA-TLX) [16].

### 2.3. Procedures and Equipment

After reviewing the inclusion/exclusion criteria, all participants were assessed. They performed two tasks (cube sorting task and gridlock puzzle), both using MT and VR, followed by the application of the SUS and NASA-TLX instruments. The stroke group performed the tasks using the nonplegic/paretic upper limb, as recommended in mirror therapy [9, 11]. The stroke-free older adults and young controls performed the tasks using the dominant upper limb/hand. The assessment protocol typically lasted an hour (the assessment could be extended according to the needs of each participant).

**Figure 1:**
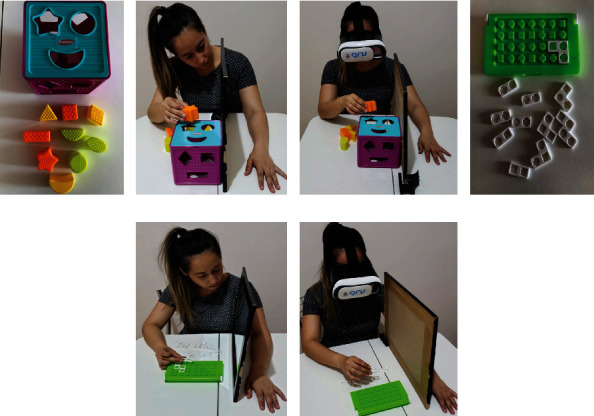
Tools used in this study to perform the cube sorting task and gridlock puzzle using conventional mirror therapy (MT) and virtual reality- (VR-) based feedback. (a, c) show the geometric shape-sorting cube (Form Fitter, Playskool, Brazil) used for the cube sorting task. (d–f) show the gridlock puzzle (Cilada, Estrela, Brazil) used for the gridlock puzzle task. (b, e) show the tools used in conventional MT, while (c, f) show the tools used in VR-based feedback.

In the MT setup, participants sat at a table with a mirror placed perpendicularly in front of them. The affected arm was hidden behind the mirror, while the unaffected hand was in front of the reflective side of the mirror. Participants were instructed to observe only the unaffected hand through the reflection in the mirror while performing tasks (Figures 1(b) and 1(e)). The VR was implemented using a smartphone app developed in the Virtual Reality Lab at PUCRS. The app provided a reversed image of the body laterality (available here Supplementary video). Unlike MT, the app provided an environment in which participants were unable to observe the real, unaffected hand performing the task. The smartphone with the working app was attached to low-cost VR goggles (Figures 1(c) and 1(f)).

Both tasks required handling objects (Figures 1(a)–1(f)). In the first task, the participants had to fit geometric figures into the correct openings of a geometric shape-sorting cube (Form Fitter, Playskool, Brazil). In the second, a gridlock puzzle game with 12 pieces was used (Cilada, Estrela, Brazil). The game has 50 possible puzzles, and we used puzzle number 25—intermediate difficulty level. For each task, the participants were instructed to fit as many pieces as they could in 5 min. The tasks were performed twice—using MT and VR, in a semirandomized sequence. After finishing both tasks with MT or VR, the participants reported their perceived usability and task load using the SUS and NASA-TLX instruments, respectively. The system usability scale (SUS) is a 10-question instrument that evaluates usability with 5 possible answers: strongly agree, agree, neutral, disagree, and strongly disagree. The score was calculated as previously described [15, 17]. The NASA task load index (NASA-TLX) evaluates workload and consists of six subscales: three of which relate to mental, physical, and temporal demand, and three others designed to assess the individual's interaction with the task (including performance, effort, and frustration). Each score is given using a visual scale ranging from zero to 100, where the participants indicate their perception. The score was calculated as previously described [16, 18].

Regarding their experiences, they were also asked two direct questions (yes/no): “Did you find the tasks difficult?” and “Did you have the impression you were actually using the affected upper limb during the tasks?”. The replies were used as complementary findings.

The Fugl Meyer assessment scale (FMA) was used to evaluate the structure and function of the upper limb based on neurological examination and sensory/motor activation. The FMA assesses active control, coordination, and reflex activity of the shoulder, elbow, wrist, and hand and also evaluates pain, passive joint movement, and sensitivity. The scoring range is from 0 to 66 points [19–21]. Functional characterization of the stroke-group members was established using the self-stroke efficacy questionnaire (SSEQ-B) [22, 23], Frenchay Activities Index [24], Barthel Index [25], and poststroke quality of life questionnaire [26].

### 2.4. Statistical Analysis

Descriptive statistics were used to characterize the sample. The factors “group” (stroke, stroke-free older adults, or young) and “system” (MT or VR) were analyzed using analysis of variance (ANOVA). Tukey's post hoc test was applied when appropriate. The chi-square test was used to evaluate categorical data. The level of statistical significance was set at *p* ≤ 0.05.

### 2.5. Ethics

This research was approved by the local Research Ethics Committee (report number 2.537.387) and is in accordance with international clinical research guidelines. Before starting the data collection, all participants signed a free and informed consent form.

## 3. Results

A total of 45 participants were included in the study with no dropouts. The sample consisted of stroke survivors, stroke-free older adults, and young adults, with a majority of males in the stroke group and females in the control groups. The majority of strokes were ischemic, and left-side body impairment was more common. The time since stroke onset varied from 4 to 348 months, and 40% of participants had experienced two or more strokes, representing a typical stroke survivor sample. The baseline Fugl Meyer assessment scale score for the stroke group was 34.00 ± 19.72 points, indicating moderate upper-limb impairment in most stroke survivors. See Table 1 for details.

Table 2 presents the results of the perceived usability, cognitive workload, and performance outcomes during the tasks completed using the MT and VR systems. The SUS scores for both systems were below the 68-cutoff point, indicating low usability. There were no significant differences in perceived usability between the two systems, but the participants reported greater difficulty in performing the tasks using the VR system, which was reflected in their performance. The young control group performed faster than the stroke and stroke-free older adult groups using both systems. Notably, when using the conventional MT system, some participants, particularly among the stroke and stroke-free older adult groups, diverted their attention from the mirror feedback to their unaffected hand. All groups reported motion sickness and visual discomfort when using the VR system. A significant percentage of participants answered “yes” to the question “Did you find the tasks difficult?” (42.2%), with stroke survivors having the highest percentage (53.3%). Furthermore, 46.6% of participants answered “yes” to the question, “Did you have the impression you were using the affected upper limb during the tasks?”, with the stroke survivor group having the lowest percentage (13.3%).

The two-way ANOVA was performed to investigate the relationship between the SUS and two factors, “group” and “feedback-based system type”. The results indicate that there was no significant interaction between the two factors (*F*_2,44_ = 2.39/*p* = 0.10) or any significant difference in the feedback-based system type (*F*_1,44_ = 1.25/*p* = 0.26). However, a significant difference was found between the studied groups (*F*_2,44_ = 6.25/*p* = 0.03). Specifically, the stroke group had significantly lower SUS scores when compared to the stroke-free young controls (*p* = 0.02). The results of the NASA-TLX analysis show there was no significant interaction between “group” and “type of feedback system” (*F*_2,44_ = 0.93/*p* = 0.91). However, a significant difference was found between the two feedback systems (*F*_1.44_ = 8.59/*p* = 0.04), with a trend towards a group effect (*F*_2,44_ = 2.85/*p* = 0.06). These findings suggest there is no difference in perceived usability between the MT and VR systems.

The analysis of the time taken to complete the cube sorting task revealed significant effects for the interaction between “group” and “type of feedback system” (*F*_2,44_ = 21.55/*p* = 0.0001), as well as for the “group” (*F*_2,44_ = 34.12/*p* = 0.0001) and “type of feedback system” (*F*1, 44 = 74.72/*p* = 0.0001) factors.

**Figure 2:**
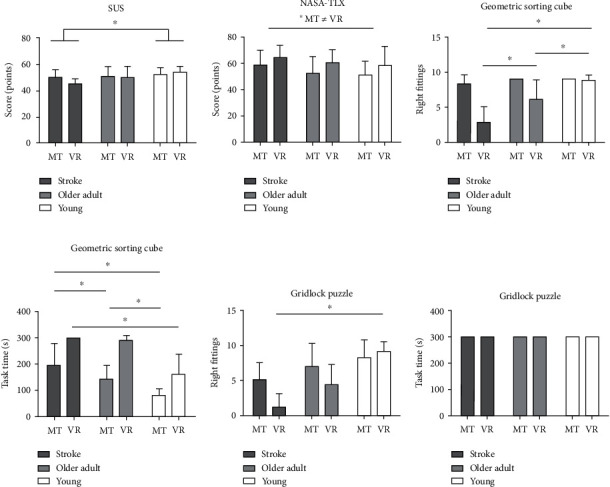
Between-group performance using MT and VR in the studied tasks. MT: conventional mirror-therapy; VR: immersive virtual mirror-therapy. ^∗^Between-group difference at *p* ≤ 0.05.

In the gridlock task, none of the groups were able to complete the activity within the allotted time, and therefore, no time-related main effects were found. However, when considering the number of pieces fitted in the gridlock board, significant effects were observed for the interaction between “group” and “type of feedback system” (*F*_2.44_ = 7.40/*p* = 0.001), as well as for the “group” (*F*_2,44_ = 37.01/*p* = 0.0001) and “type of feedback system” (*F*_1.44_ = 12.90/*p* = 0.0001) factors.  Figure 2 shows the between-group performance in the studied tasks using MT and VR.

## 4. Discussion

The current study is aimed at investigating the perceived usability of two feedback-based systems for poststroke rehabilitation: conventional mirror therapy (MT) and immersive virtual reality mirror therapy (VR). The results showed both systems had similar, but low, perceived usability. However, VR was found to be more challenging for older adults, and stroke survivors reported greater effort and less positive experience with VR. Neither task used in the study was found to be sufficiently entertaining, which may have contributed to the perceived low usability.

Virtual reality has the potential to provide a wide range of experiences that minimize distractions, highlight preferred stimuli, and facilitate motor learning and neuroplasticity [27–30]. Virtual reality goggles can also create a complete body laterality inversion, providing a more effective illusion than conventional MT [31–33]. There is evidence to suggest the illusion of using the affected limb provided by VR is able to recruit the motor cortex (M1) area in the brain, which may facilitate stroke recovery [33].

Although VR is not commonly used for mirror therapy, it has the potential to enhance immersion and improve the user experience [31, 32], making it an intriguing possibility for future research. For example, VR-embedded mirror-therapy strategies can be very useful to provide simulated real-life scenarios, in a controlled environment to help people overcome their arm/hand functioning [9, 12]. It can also be used to facilitate neurorehabilitation by providing interactive exercises that are tailored to a person's specific needs [9, 12]. Additionally, using VR-embedded mirror therapy may be utilized for pain management in phantom limb syndrome or by distracting patients from their pain or providing alternative sensations that can reduce their discomfort [10–12]. As the technology continues to improve, the potential for VR in healthcare and therapy is vast and exciting.

When selecting a virtual reality activity, it is essential to consider the characteristics of the goggles. In this study, VR-embedded mirror-therapy-induced motion sickness, dizziness, headaches, and visual-related discomfort need to be considered when prescribing rehabilitation exercises using VR. The small visual field, lens quality, and adjustment may contribute to reported visual discomfort and impact the therapeutic effectiveness of the activity [34]. However, some medications may increase tolerance for these undesirable effects and can be discussed with a physician on a case-by-case basis [35–37].

Although VR-dedicated devices generally provide a more immersive VR experience compared to smartphones with VR goggles, it is important to note that the current findings may not necessarily apply to the former. It is important to note that in developing regions, people are more likely to purchase multiuse smartphones rather than dedicated VR devices due to economic reasons. As a result, we understand that investigating the potential and limitations of using smartphones with VR capabilities for stroke rehabilitation purposes remains valuable and necessary. Further research is necessary to evaluate this influence properly and develop new low-cost technologies to alleviate VR-related discomfort. Such technologies can provide enjoyable, engaging, and motivating options for individuals recovering from stroke [11, 12].

The current findings suggest VR may pose a greater challenge for older adults compared to MT, which could be attributed to their lower frequency and familiarity with virtual technology in their daily lives [27, 28]. Stroke survivors reported greater effort when using virtual games compared to age-matched stroke-free individuals, and those with more severe impairments had a less positive experience with VR [29]. However, it is important to consider that the entertainment value of any game can vary among individuals [30], which may have contributed to the perceived low usability of both MT and VR in this study.

Interestingly, only a small proportion of stroke survivors reported feeling movement in the affected upper limb/hand using feedback-based systems, whereas nearly half of the overall sample reported feeling movement in the contralateral upper limb/hand. This finding suggests that the daily frustration of recognizing the inability to use the affected limb may inhibit belief in any functional movement in that limb, or it may be a brain-related response after stroke, which requires further investigation [3–5]. In addition to the psychological effects, it is also possible that the physical impairments resulting from stroke could contribute to the lack of perceived movement in the affected limb. For example, muscle weakness or spasticity in the affected limb could make it difficult to generate even small movements, which may not be properly perceived [4, 5, 9]. Therefore, it is important for future research to explore the various factors that may contribute to the low reported rates of movement perception in the affected limb, in order to develop more effective interventions to improve poststroke recovery outcomes.

The study's main limitation was that the stroke participants had left hemisphere impairment, resulting in right arm impairment. Consequently, participants performed the task with their nonimpaired arm, which, for most right-handed individuals, is their nondominant arm. This may have introduced a bias in the study, as performing tasks with the nondominant arm can be more challenging. Additionally, stroke survivors may experience deficits in their nonimpaired limbs, potentially further compromising their performance. Moreover, the lack of a comprehensive cognitive assessment is an additional limitation, as stroke is a risk factor for cognitive impairment [34, 38]. The low perceived usability in the stroke group may also be influenced by impairment in some cognitive domains, which requires further investigation. Overall, these limitations suggest further research to evaluate the influence of VR-related discomfort and generate new solutions to reduce it, which can provide enjoyable, engaging, and motivating options for people recovering from stroke.

## 5. Conclusion

In conclusion, the study's findings demonstrate that both MT and VR have low perceived usability, but VR seems to be more challenging, which is an important factor to consider when prescribing rehabilitation exercises. The study highlights the perceived usability features of a VR-embedded mirror therapy feedback system that uses a smartphone with a virtual reality headset, thus providing valuable insights into new directions for developing enjoyable, engaging, and motivating options to aid individuals in their recovery from a stroke.

## Figures and Tables

**Table 1:** Characterization of the studied sample.

Variable	Stroke (*n* = 15)	Older adults (*n* = 15)	Young	*p* value
Age (mean ± SD)	64.73 ± 13.01	67.33 ± 6.60	26.60 ± 3.39	0.0001^∗^
Gender (%, *n*)				
Male	73.33 (11)	20 (3)	20 (3)	0.002^∗∗^
Stroke etiology (%, *n*)				
Ischemic	93.33% (14)	—	—	—
Affected hemibody (%, *n*)		—	—	—
Left	66.66 (10)	—	—	—
Poststroke time (min–max, months)	4–348	—	—	—
Multiple strokes (two or more, %)	40%	—	—	—
Fugl-Mayer (upper limb score)	19.40 ± 11.31	—	—	—
Fugl-Mayer (wrist score)	3.33 ± 3.72	—	—	—
Fugl-Mayer (hand score)	8.87 ± 4.67	—	—	—
FIGU-Mayer (coordination and speed score)	2.40 ± 2.16	—	—	—
Fugl-Mayer (total motor function score)	34.00 ± 19.72	—	—	—
Fugl-Mayer (sensitivity score)	6.53 ± 3.87	—	—	—
Fugl-Mayer (passive movement score)	16.33 ± 8.13	—	—	—
Fugl-Mayer (joint pain score)	16.60 ± 8.10	—	—	—
SSEQ-B scale (points)	22.6 ± 9.7	—	—	—
Frenchay activities index (points)	37.2 ± 12.3	—	—	—
Barthel index (points)	77.3 ± 18.7	—	—	—
Stroke QoL questionnaire (points)	153.1 ± 28.0	—	—	—

Abbreviations. QoL: quality of life. SSEQ-B: self-stroke efficacy questionnaire–Brazilian version.

**Table 2:** Presents data on the usability perception of the conventional mirror therapy (MT) and immersive virtual reality mirror therapy (VR) systems, stratified by participant groups. The table includes information on perceived usability, cognitive workload, and performance outcomes during tasks completed using both systems.

Variable	Stroke (*n* = 15)			Older adults (*n* = 15)			Young (*n* = 15)		
	MT	VR	*p* value	MT	VR	*p* value	MT	VR	*p* value
Usability perception and performance outcomes									
SUS (score)	50.0 ± 5.7	44.83 ± 3.9	0.07	50.7 ± 7.5	49.8 ± 8.5	0.78	52.2 ± 5.2	53.8 ± 4.6	0.36
NASA-TLX (score)	58.8 ± 11.25	64.49 ± 9.2	0.14	52.56 ± 12.7	60.72 ± 9.75	0.06	51.2 ± 10.6	58.5 ± 14.2	0.12
Sorting cube (*n*° of fittings)	8.3 ± 1.2	2.8 ± 2.3	0.0001^∗^	9.0 ± 0.01	6.13 ± 2.7	0.0001^∗^	9.0 ± 0.01	8.8 ± 0.77	0.32
Sorting cube (task time, s.)	195.5 ± 82.5	300.0 ± 0.0	0.0001^∗^	141.7 ± 54.2	291.20 ± 17.9	0.0001^∗^	80.6 ± 24.8	161.7 ± 75.0	0.0001^∗^
Number of fittings (mean ± SD)	5.1 ± 2.4	1.20 ± 1.9	0.0001^∗^	7.0 ± 3.3	4.4 ± 2.8	0.03^∗^	8.27 ± 2.5	9.13 ± 1.4	0.26
Gridlock puzzle (task time, s.)	300.0 ± 0.0	300.0 ± 0.0	1.0	300.0 ± 0.0	300.0 ± 0.0	1.0	300.0 ± 0.0	300.0 ± 0.0	1.0
Looked all time in the mirror (%, *n*)	6.7 (1)	—	—	53.3 (8)	—	—	73.3 (11)	—	—
Discomfort report									
Nausea/dizziness (%, *n*)	6.7 (1)	40.0 (6)	0.03^∗^	0 (0)	20.0 (3)	0.07	0 (0)	46.7 (7)	0.003^∗^
Visual discomfort (%, *n*)	0 (0)	26.7 (4)	0.03^∗^	0 (0)	33.3 (5)	0.01^∗^	0 (0)	53.33 (8)	0.001^∗^
Headache (%, *n*)	0 (0)	6.7 (1)	0.30	0 (0)	0 (0)	1.0	0 (0)	33.3 (5)	0.01^∗^
Fatigue VR (%, *n*)	0 (0)	0 (0)	1.0	0 (0)	6.7 (1)	0.30	0 (0)	26.7 (4)	0.03^∗^
Verbalized irritation (%, *n*)	0 (0)	20 (3)	0.07	0 (0)	6.7 (1)	0.31	0 (0)	6.67 (1)	0.31
Anxiety/anguish/tension (%, *n*)	0 (0)	0 (0)	1.0	0 (0)	0 (0)	1.0	0 (0)	0 (0)	1.0
Verbalized dissatisfaction (%, *n*)	0 (0)	0 (0)	1.0	0 (0)	6.67 (1)	0.31	0 (0)	0 (0)	1.0
Discomfort with the goggles (%, *n*)	—	0 (0)	—	—	13.3 (2)	—	0 (0)	0 (0)	1.0
Other discomforts (%, *n*)	0 (0)	13.3 (2)	0.14	0 (0)	6.67 (1)	0.31	0 (0)	13.3 (2)	0.14
Requested activity interruption (%, *n*)	0 (0)	13.3 (2)	0.14	0 (0)	13.3 (2)	0.14	0 (0)	0 (0)	1.0

MT: mirror therapy; VR: immersive virtual reality mirror therapy; SD: standard deviation.

## Data Availability

The data used to support the findings of this study are available from the corresponding author upon request.

## Supplementary Materials

Supplementary Materials Supplementary Video: gridlock puzzle task (Cilada, Estrela, Brazil) performed using VR (Supplementary Materials).
