# Supplementary material for: Conventional Mirror Therapy versus Immersive Virtual Reality Mirror Therapy: The Perceived Usability after Stroke
Source: Stroke Res Treat. 2023 May 25;2023:5080699. doi: 10.1155/2023/5080699 (PMC10234727; doi:10.1155/2023/5080699)
Supplement: Supplementary Materials — Supplementary Video: gridlock puzzle task (Cilada, Estrela, Brazil) performed using VR (Supplementary Materials). [file 5080699.f1.docx]

Supplementary Video

Available for download at:

https://brpucrs-my.sharepoint.com/:v:/g/personal/10083738_pucrs_br/EQwsSqCfpxpBs7gSmYxsiUcBIIvl2KbvnXFMZ8dn7fIyzw?e=5BCHYs
